# 1036. Comparison of the hemodynamic parameters of two external chest compression devices (LUCAS versus AUTOPULSE) in a swine model of ventricular fibrillation

**DOI:** 10.1186/2197-425X-2-S1-P83

**Published:** 2014-09-26

**Authors:** C Pantazopoulos, I Floros, A Mega, C Rigas, I Pavleas, P Vernikos, N Archontoulis, D Xanthis, N Iacovidou, T Xanthos

**Affiliations:** Intensive Care Unit, General Hospital of Athens Laiko, Athens, Greece; Medical School, Neonatology, University of Athens, Athens, Greece; Medical School, M.Sc. Programme in Cardiorespiratory Resuscitation, University of Athens, Athens, Greece

## Introduction

Given the difficulty of performing efficient CPR compressions, technology has turned to automaticity. LUCAS device has a pneumatically driven piston to compress the heart and uses active decompression suction on the upstroke. AUTOPULSE is a load distributing band compressor, that is mechanically actuated and battery driven. It provides both direct compression and semi-circumferential thoracic compression.

## Objectives

To compare 2 different external chest compression devices (LUCAS and AUTOPULSE) regarding the hemodynamic effects during cardiorespiratory resuscitation.

## Methods

Forty (40) pigs were randomly allocated into 2 groups. Group L (LUCAS), n=20 and Group A (AUTOPULSE), n=20. After anesthesia ventricular fibrillation was induced. Five minutes post cardiac arrest without treatment, resuscitation was initiated. Electrocardiography, intra-arterial pressure (carotid artery) and Swan-Ganz catheter were used to monitor central venous pressure, cardiac output, cardiac index, systemic vascular resistance and pulmonary vascular resistance prior to ventricular fibrillation and during resuscitation in both groups.

## Results

The hemodynamic parameters demonstrated that there is no statistical difference in mean arterial pressure between the 2 devices but there was statistically significant difference (P< 0.05) in the cardiac output with LUCAS generating higher values than AUTOPULSE.

## Conclusions

The mean arterial pressure that is produced by the 2 devices is similar, while the cardiac output produced by LUCAS is higher than AUTOPULSE.
